# Static magnetic field reduces cisplatin resistance via increasing apoptosis pathways and genotoxicity in cancer cell lines

**DOI:** 10.1038/s41598-024-56605-1

**Published:** 2024-03-09

**Authors:** Jaber Zafari, Nima Rastegar-Pouyani, Fatemeh Javani Jouni, Nabaa Najjar, Seyedeh Zohreh Azarshin, Emad Jafarzadeh, Parviz Abdolmaleki, Farshad Hoseini Shirazi

**Affiliations:** 1 Department of Pharmacology and Toxicology, Faculty of Pharmacy, Tehran Medical Sciences, Islamic Azad University, Tehran, Iran; 2 Department of Pharmacology and Toxicology, Faculty of Pharmacy, Tehran University of Medical Sciences, Tehran, Iran; 3 Department of Biochemistry and Biophysics, Faculty of Advanced Sciences and Technology, Tehran Medical Sciences, Islamic Azad University, Tehran, Iran; 4 Laser Application in Medical Sciences Research Center, Shahid Beheshti University of Medical Sciences, Tehran, Iran; 5 Department of Biophysics, Faculty of Biological Sciences, Tarbiat Modares University, Tehran, Iran; 6 Pharmaceutical Sciences Research Center and Department of Toxicology and Pharmacology, Faculty of Pharmacy, Shahid Behesthi University of Medical Sciences, Tehran, Iran

**Keywords:** Cisplatin, Static magnetic field, Ovarian epithelial cancer, Apoptosis, Cancer, Cell biology

## Abstract

Cisplatin is a chemotherapy drug widely used in cancer treatment. Alongside its clinical benefits, however, it may inflict intolerable toxicity and other adverse effects on healthy tissues. Due to the limitation of administering a high dose of cisplatin as well as cancer drug resistance, it is necessary to utilize new methods optimizing treatment modalities through both higher therapeutic efficacy and reduced administered doses of radiation and drugs. In this study, sensitive (A2780) and resistant (A2780CP) ovarian carcinoma cells underwent treatment with cisplatin + static magnetic field (SMF). First, the levels of genotoxicity after treatment were evaluated by Comet assay. Then, cell cycle analysis and apoptosis assay were conducted by a flow cytometer. Lastly, the expression levels of genes involved in apoptosis and cellular drug uptake were investigated by PCR. After treating different groups of cells for 24, 48, and 96 h, the co-treatment of SMF and cisplatin as a combination managed to increase the amount of DNA damage in both sensitive and resistant cell lines. A considerable increase in mortality of cells was also observed mostly in the form of apoptosis, which was caused by inhibition of the cell cycle. The combination also increased the expression levels of apoptotic genes, namely P53 and P21; however, it did not have much effect on the expression levels of BCL2. Besides, the levels of CTR1 gene expression increased significantly in the groups receiving the aforementioned combination. Our study suggests that the combination of cisplatin + SMF might have clinical potential which needs further investigations through future studies.

## Introduction

As one of the leading causes of death worldwide, cancer has long posed a threat to human health. Despite the adoption of conventional cancer therapies, including surgery, chemotherapy, radiation therapy, and immunotherapy, among others, none has been able to overwhelm the menace of cancer for good as most of the mono or combination therapies eventually lead to intolerable adverse effects and/or cancer resistance^1^. Indeed, due to the annually increasing number of cancer patients, the need for new anticancer approaches has never been greater^2,3^.

Cisplatin is a chemotherapy drug that acts through interfering with DNA replication and is mainly administered for the treatment of solid tumors including ovarian, testicular, and lung cancer, similar to many other anticancer agents, it mostly targets cells with a high proliferation index^4^. Given the notorious adverse effects of cisplatin such as nephrotoxicity and neurotoxicity, reducing its administrated dose through combination therapy with other modalities and/or other drugs is highly warranted^5,6^. In addition, although initial responsiveness to cisplatin is desirably high, unfortunately, many patients will ultimately face cancer relapse due to cisplatin resistance, which is all the more reason to find and employ novel combinations of cisplatin and other agents of interest^7^. In our previous In vitro study, we found that the combination of cisplatin and static magnetic fields (SMF) had substantially increased the efficacy of treatment by reaching lower IC_50_ values of cisplatin alongside higher levels of cisplatin uptake by cancer cells^8^.

All organisms are exposed to magnetic fields (MFs) from different sources on a daily basis; therefore, a better understanding of the inevitable influence of MF on living beings, including humans, has been of great importance^9^. Moderate Static magnetic fields (SMFs) with intensity ranging from 1 mT to 1 T have been found to significantly affect a variety of biological systems^10^. On the one hand, several studies have demonstrated the possibly deleterious effects of such an exposure, while on the other hand, there have been other studies reporting piquant implications regarding the clinical utility of MFs, which may in the long run yield a whole new modality against medical conditions such as cancer in future^11–13^. A study by Bekhite et.al reported that SMF could improve cardiomyocyte differentiation of mouse embryonic stem cells which interestingly led to higher generation rates of cardiomyocytes without any genetic or pharmacological interventions^14^. Besides, there is intriguing evidence pointing to the anti-cancer effects of MFs^15–17^. Indeed, by altering the distribution of membrane receptors and transmembrane ion fluxes, and increasing ROS and P53 levels, among others, MFs have far-reaching effects on cancer cells and their viability^11,18,19^. A study by Vergallo et al. reported that, while SMF could not impact the viability of human neuroblastoma SH-SY5Y Cells, it surprisingly elevated the production of ROS and changed the morphology of cells^20^. Moreover, it has been shown that SMFs are also able to upregulate the expression levels of certain genes among microorganisms^21^. It has been reported that a magnetic field of low frequency was able to increase the efficiency of DNA repair, which was found to be mediated by the induced overproduction of heat shock proteins DnaK/J (Hsp70/40)^22^. Considering such functional aspects, and as mentioned above, many studies have recently dedicated to reducing the administrated doses of chemo drugs e.g., cisplatin by harnessing MFs in a new combination, which might result in lower risks of normal tissue toxicity and other adverse effects.

Following on from our previous study^8^ and based on its results, this study was to be conducted in order to get new perspectives on the effects of the combination of cisplatin + SMF. First, cell cycle analysis and apoptosis assay were performed on both A2780 (human ovarian carcinoma-sensitive to cisplatin) and A2780-CP (human ovarian carcinoma-resistant to cisplatin) cell lines after being treated with the combination. Then, as inductively coupled plasma (ICP) in our previous study had reported increased cisplatin uptake following applying the combination, comet assay was to be performed to get a better grasp of how the higher levels of cisplatin uptake would perhaps drastically damage the DNA compared to cisplatin alone. Lastly, the fold changes in expression levels of some of the well-known genes involved in apoptosis, including P_53_, P_21_, and Bcl_2,_ as well as CTR1 governing cisplatin uptake were analyzed. Lastly, it should be mentioned the present research involving cancer cell lines adhered to ethical guidelines.

## Materials and methods

### Cell culture

A2780 and A2780-CP cell lines—respectively sensitive and resistant—were purchased from the National Cell Bank of Iran (NCBI). These cells were considered as passage zero and treatments and tests were performed on the second passage. The cells cultured at 37 °C, 5% CO2 in RPMI-1640 medium (Gibco) supplemented with 10% fetal bovine serum (Gibco), 100 μg/mL of streptomycin and 100 U/mL of penicillin (P/S; Gibco). Upon reaching 70–80% confluency, cells were to be passaged and plated at 1:2 or 1:3 dilutions every 3–4 days using 0.25% trypsin and 1mM EDTA (Invitrogen LT, Merelbeke, Belgium).

### Static magnetic field application

**Figure 1:**
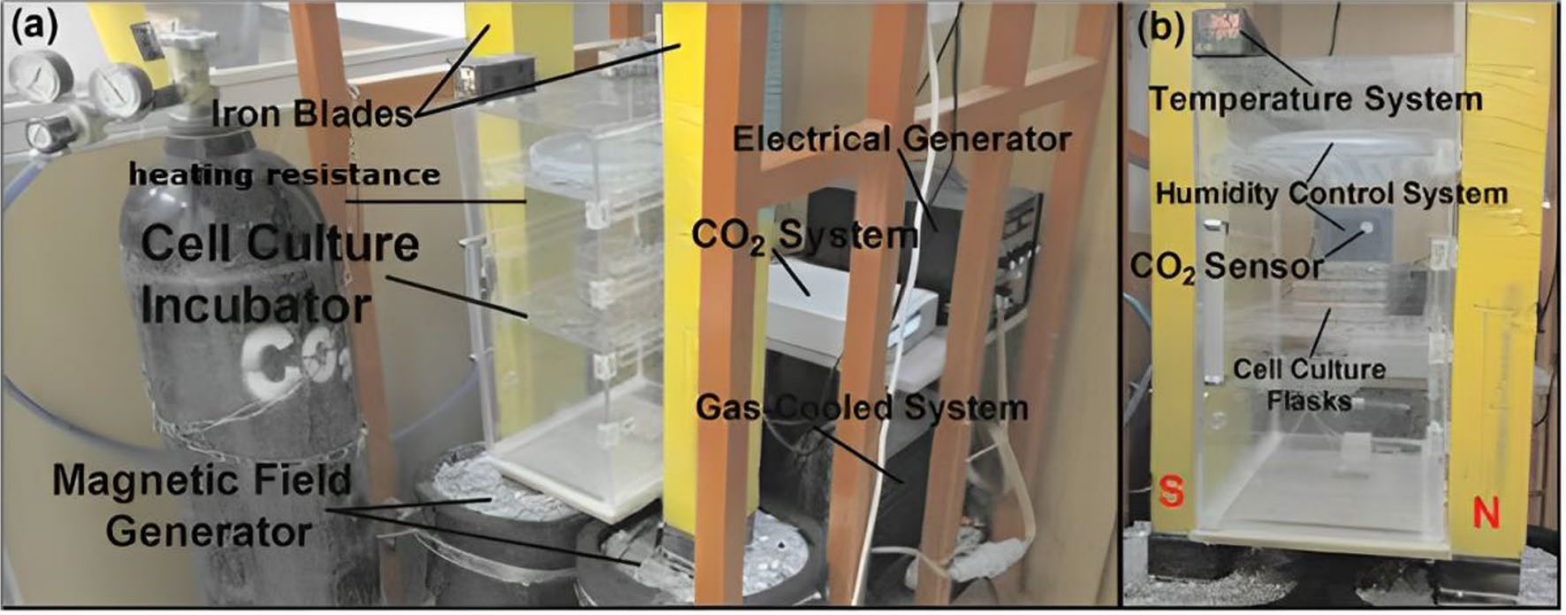
Illustration of magnetic field exposure system. (**a**) The whole body of the exposure system. (**b**) The movable plexiglass incubator (exposure unit) within iron blades provides standard conditions of cell culture^23^.

SMF exposure was conducted via a local generator, which consists of two wire coils with 3.0 mm in diameter and inductance of 2 H, resistance of 3 Ω, and a heat resistance of up to 200 °C. The wire length of each coil was approximately 1 km and each coil weighed about 40 kg. The system was also supplied with a prismatic chamber (23 × 20 × 50 cm^3^) incubator utilizing various sensors to check the temperature and humidity of the air encompassing each flask, and the CO_2_ pressure. A gas chiller with optimal control of temperature was used in order to cool down the system, which includes an engine, an evaporator, a condenser, and a refrigerant gas. It should be mentioned that the aforementioned engine is remote from the exposure unit to avert any undesirable SMF interventions. The evaporator surrounded the external side of the coils, and as a result, effectually cooled down the system. Owing to the volume of the operator employed in the present system, a one-third Danfoss motor was exploited. In addition, the condenser was to be one-third as equal to the motor. Freon-12 kind was the gas used for this motor (Fig. 1). The SMFs were directed across two iron blades (with a 1-m height and a cross-section of 10 cm^2^) by coils. The system was meant to create homogeneous SMF in the range of 0.5 mT to 90 mT in steady circumstances. To maintain the exposure system an electronic board was employed. The homogeneity of the field was 95% across the region the cells were situated, on the various shelves of the incubator. The field between iron blades was assessed by a 13,610.93 PHYWE (Gottingen, Germany) Tesla meter. The exposure times were 24, 48, and 96 h. Prior to every exposure, the SMF intensity was checked for apposite intensity (15 mT) via a Tesla meter. The electric generator used a 220V AC Power Supply in conjunction with a single-phase full-wave rectifier and a variable transformer. To create the various SMF intensities, the switching power supply was able to change the potential differences up to 50 V with a direct current up to 16 A. The circulation system included an engine, refrigerant gas, a condenser, and an evaporator, which surrounded the external side of the coils.

Shielding the apparatus from background noise is indeed an important issue in experimental setup. We took these measures to avoid the interference of background noise with our experiment setup. (1) The apparatus was located in an isolated room in which there were not any other electrical instruments. (2) The apparatus was far away from any wire-passing near it. (3) The control cells were kept far enough from the MF-producing apparatus, to avoid any potential exposure to the MF. Moreover, other electric appliances and laboratory facilities were not working; the control samples were therefore exposed only to the extremely low MF of the earth (50–60 µT), as the treatment groups were too. (4) The presence of any pulsation in the current from rectifier into the MF generating apparatus, was tested by an oscilloscope (40 MHz, model 8040, Leader, Yokohama, Japan) and a pulsation frequency of 50 Hz with a range of voltage variation about ± 1 V, was shown. The presence of this pulsation frequency may be related to the shortcoming of the single-phase full-wave rectifier, which provides a ripple voltage around (5%).This small ripple voltage suggests that the generated MF can be considered highly homogeneous. (5) An electronic board was used to stabilize the system so that we always got a uniform MF. (6) The Calibration of the system as well as tests for the accuracy and uniformity of the MFs were performed by a teslameter (13,610.93, PHYWE, Gottingen, Germany) with a probe type of Hall Sound. The accuracy of the system was ± 0.1% for MF and the range of measurements was 3 µT–30 mT.

**Figure 2:**
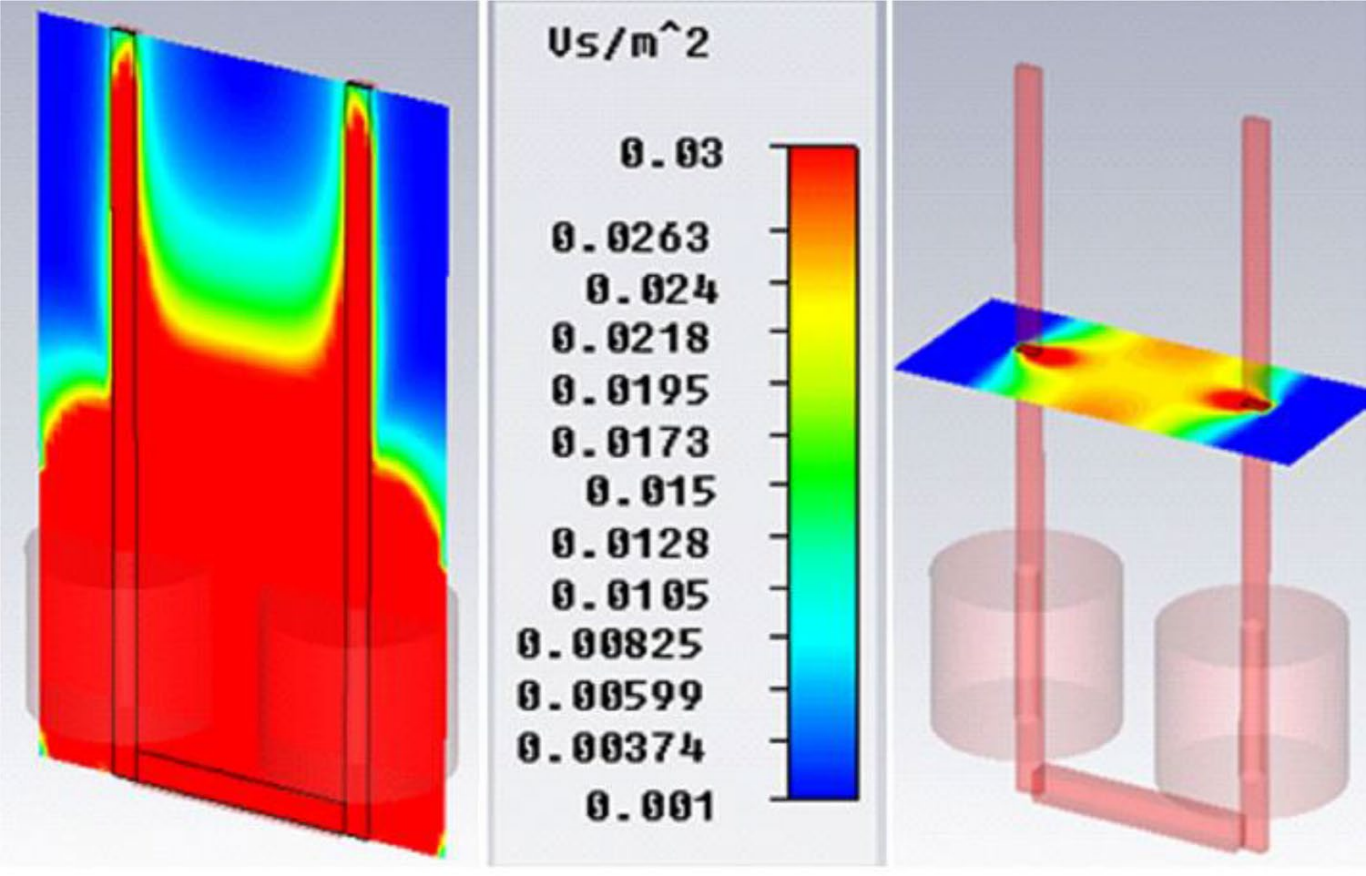
The distribution of magnetic field diagram in the iron blades and coils of exposure system. The place between two iron blades (pink axels), which is highlighted in yellow shows homogeneous magnetic field intensity^23^.

SMF was evaluated by Complete Technology for 3D Simulation (CST STUDIO 2011 software) to choose the best area for our samples across the exposure unit of the SMF-exposure system (http://www.CST.com) (Fig. 2). The profile of field emission in coils is exhibited with surfaces of the same color^23^. The value of the geomagnetic field in our laboratory was found to be 47 µT by the Tehran Geomagnetic Observatory, Institute of Biophysics, University of Tehran.

### Cisplatin and SMF treatments

Each cell type was divided into 4 groups. The first group of cells was the control group, without any treatment, the second one was exposed to the SMF (15 mT) alone without any interruption, and the third group was treated with the previously measured IC_50_ values of cisplatin by MMT assay (Table 1), and the last one was the combination group, treated with both SMF (15 mT) and drug (IC_50_). This study was carried out at three separate times (24, 48, and 96 h) with 15 mT SMF, which was found to be the optimal intensity in our previous study^8^.

**Table 1:** The IC50 values of cisplatin (μg/ml)^8^.

	24 h		48 h		96 h	
	Sensitive	Resistant	Sensitive	Resistant	Sensitive	Resistant
Cisplatin	27.69 ± 9.58	61.15 ± 13.91	3.55 ± 3.14	14.86 ± 4.8	1.72 ± 1.09	6.19 ± 1.11
Cisplatin + SMF	14.21 ± 5.19	12.75 ± 4.72	4.31 ± 3.72	4.84 ± 1.95	4.01 ± 3.54	6.41 ± 1.23

### Comet assay

After diluting the treated cells with culture medium to the final concentration of 2 × 10^5^ cells/ml, 150 μl cell suspension of each group was immediately mixed with 150 μl of low melting point (LMP) agarose (Sigma-Aldrich) and subsequently coated on a microscope slide that had been previously prepared by 1% agarose (Sigma-Aldrich). The slides were stored at 4 °C for 10 min allowing the gel to solidify. Upon being coated with a supportive layer (LMP), slides were then placed in a chilled (4 °C) lysis buffer, containing 2.5 M sodium chloride, 100 mM EDTA, 10 mM tris base, 0.01% triton x-100 with pH = 10, for 1 h. After placing the slides in the electrophoresis tank, the denaturing solution, including 300 mM NaOH, and 100 mM EDTA with PH = 12, was poured on the surface of the slides in order to unwind the nuclear DNA. First, slides were kept in the denaturing solution for 30 min, and then the electrophoresis was performed at a separation voltage of 1 V/cm for 30 min. In the next step, Slides were placed in the neutralizing buffer, including 400 mM tris buffer with PH = 7.5 for 5 min, and stained with 20 μg/lit ethidium bromide dye. Captured images by fluorescence microscope were analyzed with comet score for fluorescence intensity. To evaluate the amount of DNA damage, two factors, comet tail DNA%, and the Olive Tail Moment values, were utilized and compared to the control.

### Cell cycle analysis

The cell cycle progression of both A2780 and A2780-CP cells was evaluated by flow cytometry. Cells from different groups were harvested, rinsed with PBS, and fixed following incubation with 70% cold ethanol for 2 h at 4 °C. After centrifugation, the cell pellets were stained with the solution, including propidium iodide (PI; Sigma) with a concentration of 1 mg/ml, 20 μl DNase-free RNase A (Invitrogen), and 1μl Triton X-100 dissolved in PBS, and stored at room temperature in the dark for 30 min. DNA content was measured via a FACSCalibur flow cytometer (BD Biosciences, San Jose, CA, USA), and the results were later assessed by WIN-MDI.

### Apoptosis assay

Annexin V/PI staining was used to measure the percentage of cells undergoing cell death. Both cell lines were treated similarly to the cell cycle assay. After dissociation, cells were then resuspended in 100 μl binding buffer. Subsequently, the cell suspension was incubated with PI (2 μg/ml) and 5 μl of Annexin V-FITC (Invitrogen, USA) at room temperature for 15 min in the dark. Just before analyzing, the labeled cells were diluted again with 400 μl binding buffer.

### Gene expression analysis

The fold changes in expression levels of genes, including P53, P21, CTR1, and Bcl2 were analyzed following treatment using real-time polymerase chain reaction (PCR). RNA extraction from both cell lines was performed using RNeasy Mini Kit (Qiagen, USA) based on the instruction of the manufacturer. To synthesize cDNA from the purified RNA by reverse transcription, QuantiTect Reverse Transcription Kit (Qiagen, USA) was used. Real-time PCR was performed by ABI Step One (Applied Biosystems, Sequence Detection Systems, Foster City, CA), and Solis BioDyne kit was used to amplify cDNA fragments (5 × hot firepole EvaGreen qPCR mix plus (ROX)). To normalize the expression levels of our target gene according to the comparative 2 ^−ΔΔCt^ method, GAPDH was employed as the internal reference gene. Primers, which are shown in Table 2, were designed by the primer blast (NCBI) and evaluated for possible secondary structures with gene runner. Lastly, it should be noted that standard curves and melting curves were drawn for all primers to ensure their specificity.

**Table 2:** Primer sequences used for real-time PCR.

Gene	Product length	Primer sequences (Oligo sequence 5' → 3')	Melt temperature (°C)
P53	125	Forward: ACCTATGGAAACTACTTCCTGA Reverse: TTCATCTGGACCTGGGTCTTCA	73.66
CTR1	293	Forward: TGCCATTGACATCAAACTCTATGGC Reverse: GGCTCTAAGATGATGGAGAAGGA	70.5
Bcl2	110	Forward: GGAGGCTGGGATGCCTTTGTGGA Reverse: CAAGCTCCCACCAGGGCCAAACT	79.15
P21	188	Forward: AGAGGAGGCGCCATGTCAGAAC Reverse: AGTGGTGTCTCGGTGACAAAGT	74.56
Gapdh	117	Forward: AGGGCTGCTTTTAACTCTGGT Reverse: GCCATGGAATTTGCCATGGGT	85.87

### Statistical analysis

All of the experiments were carried out with at least three independent repetitions. Data were presented as mean ± SD (standard deviation) and were analyzed using one-way repeated measure analysis of variance (ANOVA) followed by Tukey’s post hoc test. P-values less than 0.05 were considered statistically significant.

## Results

### Genotoxicity induced by the combination of cisplatin + SMF

**Figure 3:**
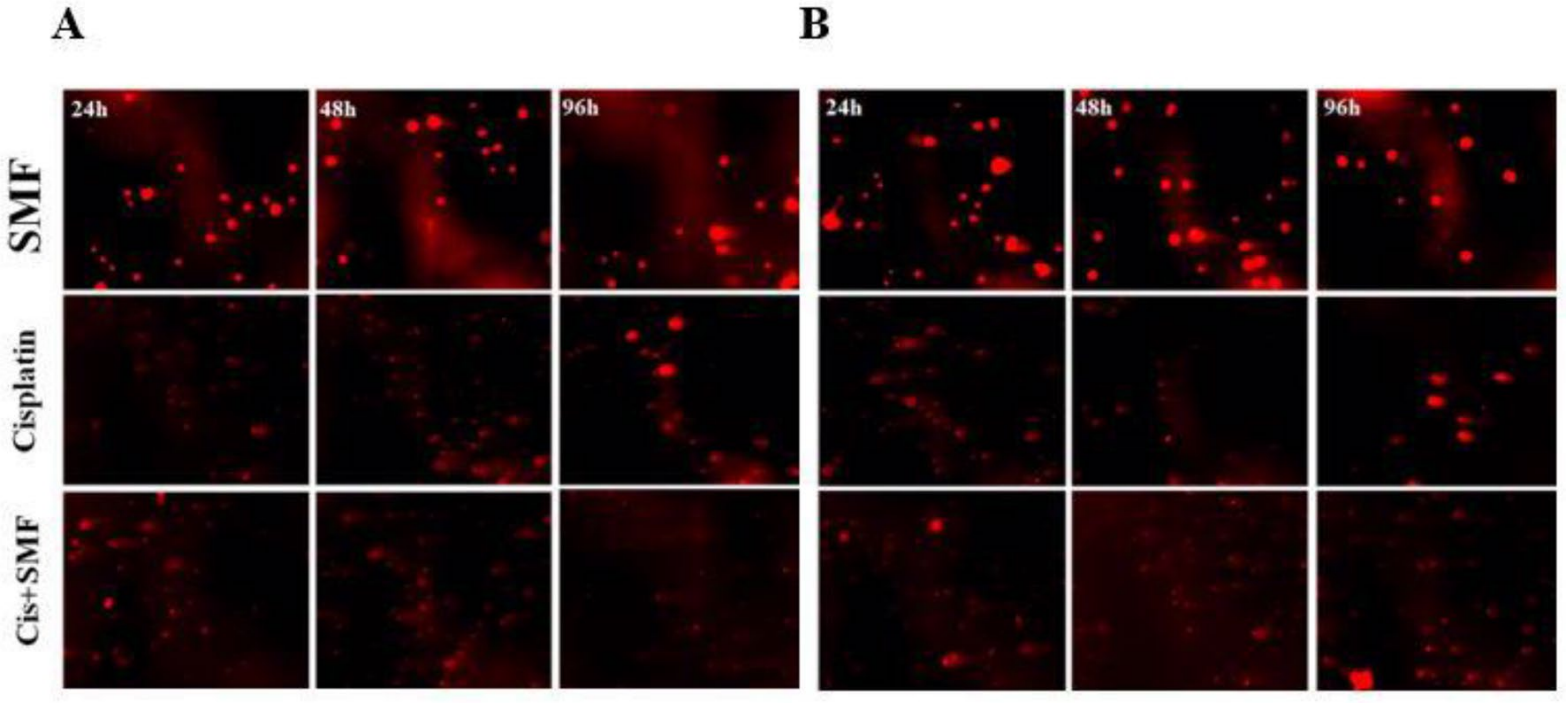
Genotoxicity under the influence of the combination of cis + SMF. (**A**) A2780CP (**B**) A2780.

**Figure 4:**
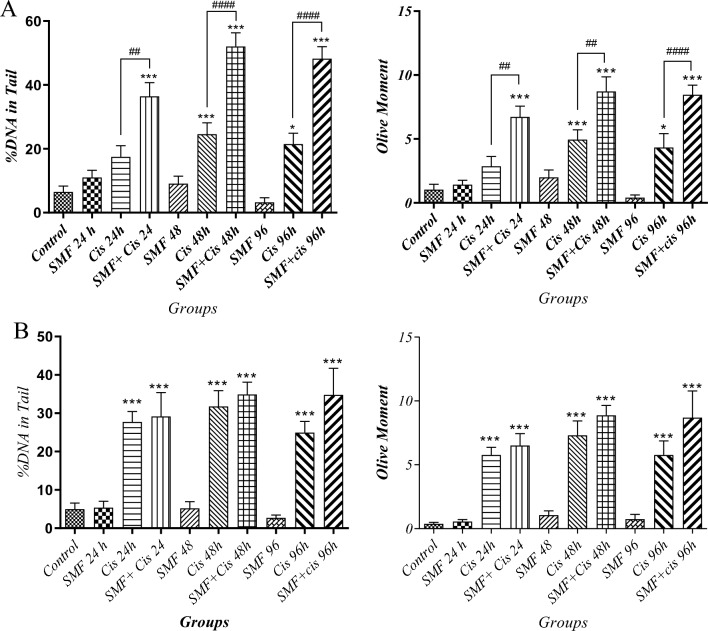
Evaluation of comet tail DNA% and the Olive Tail Moment values. Results were expressed as mean ± SD, n = 3 (*P < 0.05, ***P < 0.001, ^##^P < 0.01, and ^###^P < 0.001; *: compared to the control and #: compared to stand-alone cisplatin treatment counterpart) (**A**) A2780 (**B**) A2780CP.

**Figure 5:**
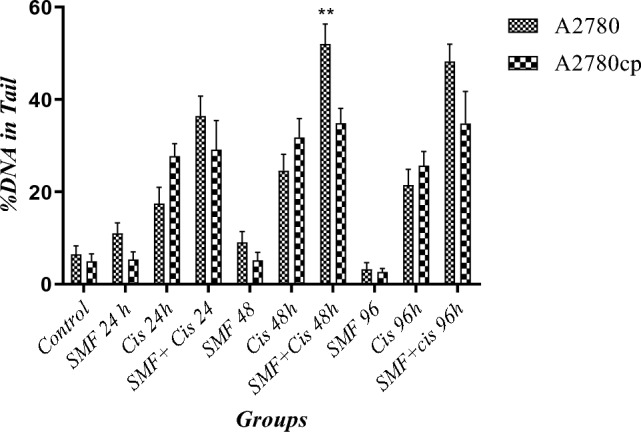
Comparative evaluation of comet tail DNA% between A2780CP and A2780 (**P < 0.01).

The images of every sample (Fig. 3), containing roughly 10 cells, were analyzed by Cometscore. As shown in Fig. 4, among sensitive cells, the comet tail DNA% and olive moment of each combination group were significantly higher compared to the control. More importantly, these increased comet tail DNA% and olive moment in combination groups of sensitive cells were significantly higher than those in their stand-alone cisplatin treatment counterparts, which suggested the synergistic effect of SMF with cisplatin resulting in a more efficacious treatment among sensitive cells. However, in resistant cells, although both comet tail DNA% and olive moment in combination groups were higher than those in both the control and their stand-alone cisplatin treatment counterparts, the difference between each combination and its cisplatin alone counterpart was not statistically significant. Moreover, the comet tail DNA% of both cell lines were comparably evaluated; surprisingly, it was the sensitive group with 48h combination therapy that had a significantly higher comet tail DNA% compared to that in its resistant counterpart, whereas no such significance was found among other groups. Taken together, the results proposed that cisplatin + SMF was able to act more efficaciously in comparison with cisplatin monotherapy in sensitive cells, while such an event was not the case in the resistant ones (Fig. 5).

### Cell cycle analysis

**Figure 6:**
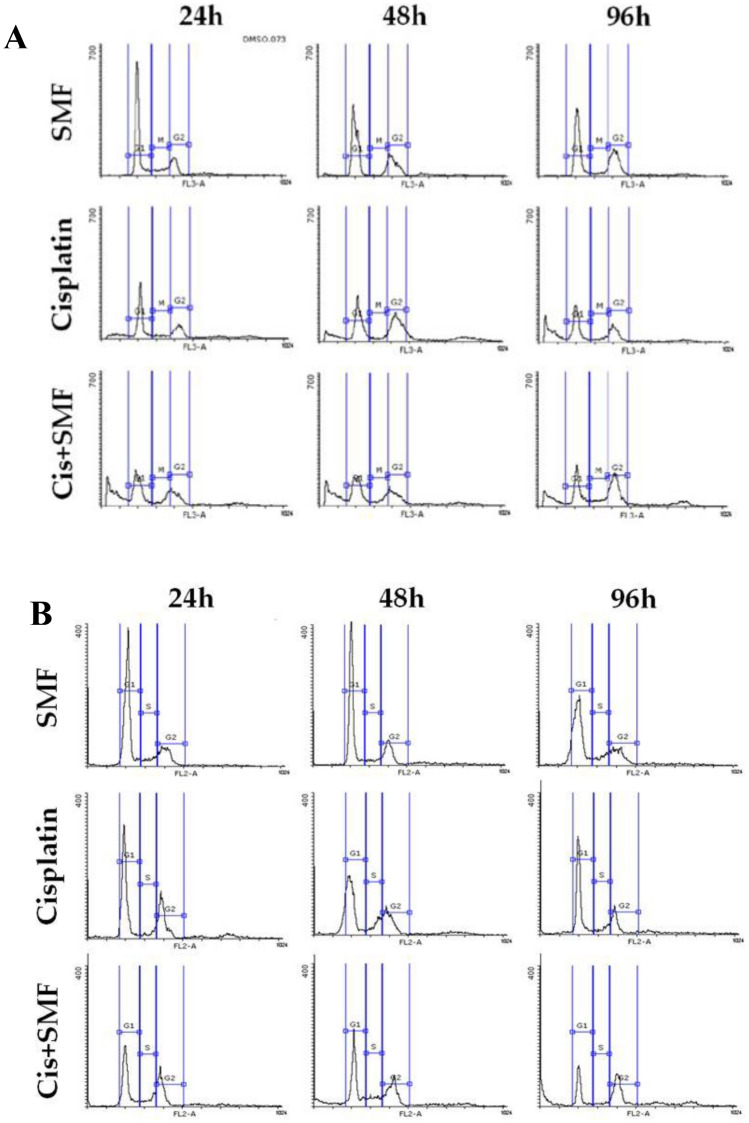
The cell cycle histograms of (**A**) A2780 (**B**) A2780CP.

**Figure 7:**
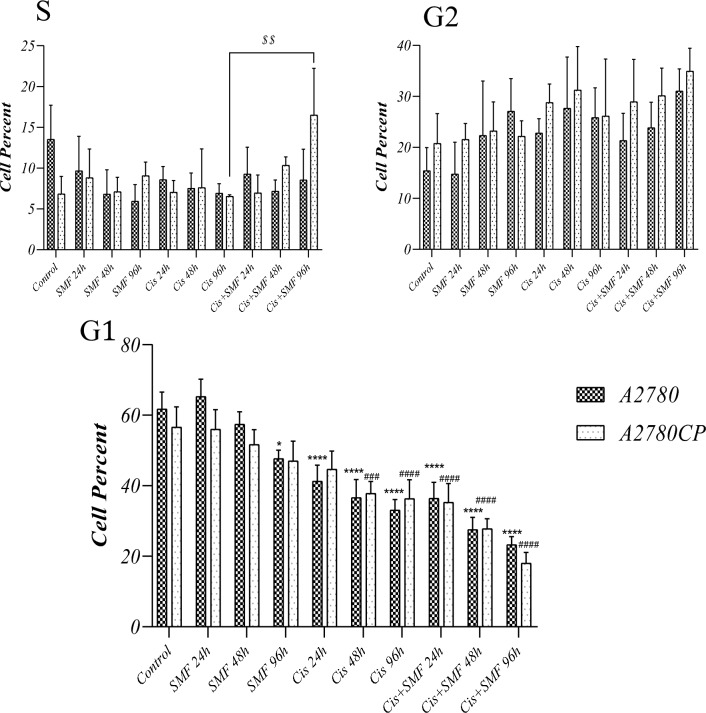
Cell cycle analysis of phases G1, S, and G2. Results were expressed as mean ± SD, n = 3 (*P < 0.05, ****P < 0.0001, ^###^P < 0.001, ^####^P < 0.0001, and ^$$^P < 0.01; *compared to the control in sensitive cells, ^#^compared to the control in resistant cells, and ^$^compared to stand-alone cisplatin treatment counterpart).

After treatment with the concentrations, the cell cycle of each group was analyzed by flow cytometry as presented in Fig. 6 and 7. Following analysis, surprisingly, it was only in the S phase that cisplatin + SMF for 96h in resistant cells resulted in a higher percentage of cells compared to its stand-alone cisplatin treatment. Besides, no significant difference was detected in the G2 phase. In the G2 phase, all groups from both cell lines treated with cisplatin or cis + SMF had higher percentages of cells compared to their controls; however, no statistical difference was found between each combination group and its stand-alone cisplatin treatment.

### Apoptosis assay

**Figure 8:**
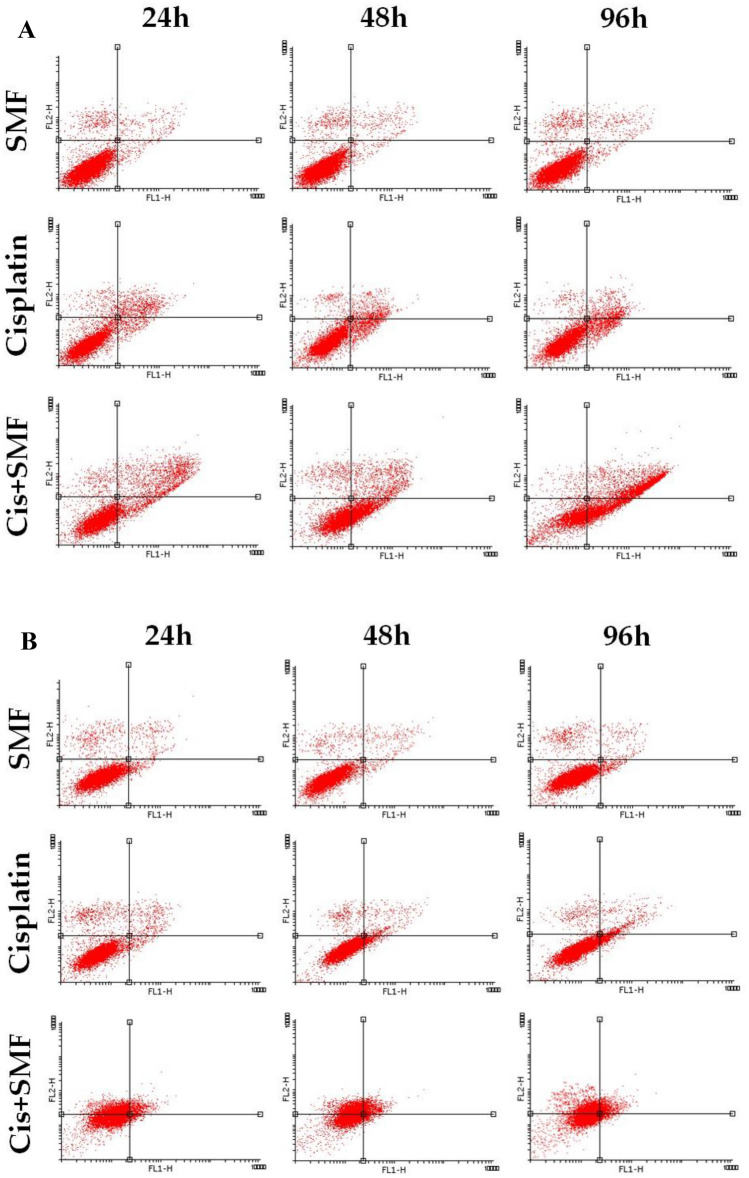
The percentages and types of cell death among (**A**) A2780 (**B**) A2780CP.

**Figure 9:**
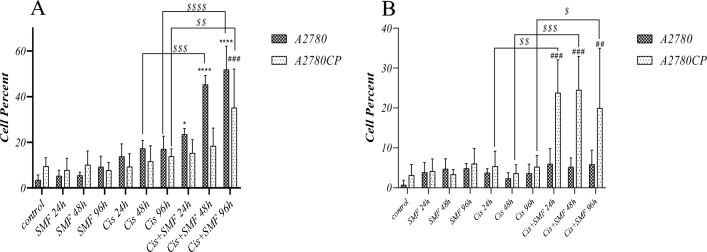
Comparative evaluation of the percentages and types of cell death (**A**) Total apoptosis (**B**) Necrosis. Results were expressed as mean ± SD, n = 3 (****P < 0.0001, ^##^P < 0.01, ^###^P < 0.001, ^$^P < 0.05, ^$$^P < 0.01,^$$$^P < 0.001, and ^$$$$^P < 0.0001; *compared to the control in sensitive cells, ^#^compared to the control in resistant cells, and ^$^compared to stand-alone cisplatin treatment counterpart).

The results showed that cis + SMF for 96h in both cell lines and cis + SMF for 48h in sensitive cells had significantly higher numbers of cells undergoing total apoptosis compared to their stand-alone cisplatin counterparts. Besides, cis + SMF for 24h in sensitive cells had higher apoptotic cells compared to its control, while was not statistically different from its cisplatin monotherapy counterpart. In the case of necrosis, interesting results were observed among resistant cells as all the combination therapy groups had higher percentages of necrotic cells compared to their stand-alone cisplatin treatment counterparts. Taken together, it seems that cis + SMF affects resistant and sensitive cells more significantly through induction of necrosis and apoptosis, respectively, in comparison with their stand-alone cisplatin treatment counterparts (Figs. 8 and 9).

### Gene expression analysis

**Figure 10:**
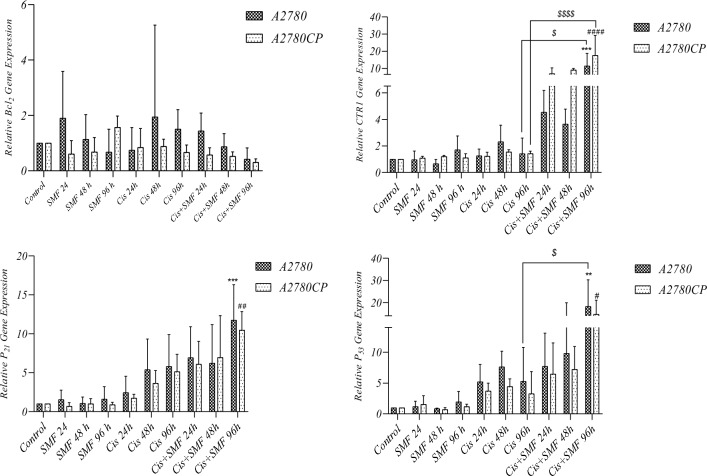
Relative mRNA expression levels of target genes, namely P53, P21, Bcl2, and CTR1, in (**A**) A2780 and (**B**) A2780CP. Results were expressed as mean ± SD, n = 3 (**P < 0.01, ***P < 0.001, ^#^P < 0.05, ^##^P < 0.01, ^####^P < 0.0001, ^$^P < 0.05, and ^$$$^P < 0.001; *compared to the control in sensitive cells, ^#^compared to the control in resistant cells, and $: compared to stand-alone cisplatin treatment counterpart).

Figure 10 represents the evaluation of expression levels in P53, P21, Bcl2, and CTR1 among both cell lines. While there were seemingly distinctions in Bcl2 fold changes in both cell lines, especially the sensitive ones, no statistical significance was found, as for CTR1, the fold changes in cis + SMF for 96h groups in both cell lines were substantially higher compared to their controls and stand-alone cisplatin treatment counterparts. Regarding p21, cis + SMF for 96h groups in both cell lines, unlike their stand-alone cisplatin treatment counterparts, had significantly higher fold changes compared to their controls. Similarly, in the case of p53, cis + SMF for 96h groups in both cell lines unlike their stand-alone cisplatin treatment counterparts, had significantly higher fold changes compared to their controls, and on top of that, the combination group in sensitive cells had a higher fold change compared to its stand-alone cisplatin treatment counterpart as well. The results proposed that cis + SMF groups in both cell lines had the highest efficacy in upregulating the expression levels of CTR1, P21, and P5.

## Discussion

During the past decades, given the daily exposure to electromagnetic fields widely existing in the environment, uncertainty has persisted concerning their possibly harmful effects on human health; as a result, many studies have strived to delineate such effects of magnetic fields from various aspects, which surprisingly provided contradictory results. Some of them report that the effect of weak magnetic fields on living systems is transient, and as the intensity of the field starts to decrease the system returns to its original state^24,25^.

Despite the recent medical advances, cancer has unfortunately remained a daunting challenge for health systems. One great hurdle in the clinical settings that has been faced by many oncologists is the emergence of severe adverse effects in patients following administration of the conventional anti-cancer modalities such as chemotherapy and radiotherapy. On top of that, in many cases, drug resistance may occur which may necessitate receiving higher doses of such narrow therapeutic index cytotoxic agents although not feasible in practice^26,27^. Therefore, given the annually increasing number of patients diagnosed with cancer, as well as a lack of definitive clinical success by the existing modalities in many cases, finding novel approaches to inhibit cancer progression and predict a better prognosis in the affected individuals are of the essence^28^.

Our previous study^8^ showed that SMF was able to increase the cytotoxic effects of cisplatin on cell viability and reduce the resistance of A2780-CP cells through the production of verruca-shaped structures at the cell surface. Moreover, there have been several experiments regarding the evaluation of the possible genotoxicity caused by SMF, most of which indicated that the magnetic field alone had no effect on genotoxicity^15^. It has been reported that low to medium intensities of SMFs do not have a significant effect on cells. However, in the presence of external factors, they can lead to the generation of free radicals thereby making their effects notably pronounced^29,30^. The co-treatment of cells with SMF and compounds such as Fecl3 has exhibited higher levels of lipid peroxidation and oxidative damage to DNA, all of which point to the synergistic effects of SMF on free radicals^15,23^. Gurhan et.al. demonstrated that, following an increase in the intensity of SMFs, oxidative stress reached somewhat higher levels, while in contrast, the levels of nitric oxide and superoxide were both dropped^31^. A study by Rageh et al. asserted that a low dose of cisplatin in conjunction with MF substantially increased malondialdehyde levels, a marker for oxidative stress, whereas reduced glutathione levels, and superoxide dismutase activity, and overall improved the effectiveness of low administered doses of cisplatin benefiting from considerably less nephrotoxicity^32^. Since the amount of iron in cancer cells is higher than that in normal counterparts, by altering intracellular iron homeostasis, the magnetic field can increase free iron within the nucleus and cytoplasm^33^. Also, the increased levels of iron can increase hydroxyl radicals through the Fenton reaction^34^. Furthermore, cisplatin produces reactive oxygen species such as superoxide anion and hydroxyl radicals^35^. An increase in reactive oxygen species causes lipid peroxidation of the membrane and eventually causes the inability of the antioxidant defense mechanism against damage caused by free radicals, hence genotoxicity^36^. Similar to the aforementioned findings from other studies and as shown in Figs. 3 and 4, no considerable genetic damage was observed in groups treated with SMF alone from both cell lines, on the other hand, both stand-alone cisplatin treatment and combination groups mostly had significantly higher comet tail DNA% and olive moment in comparison to their controls. Furthermore, comparative analyses demonstrated that, among sensitive cells, there were significant differences regarding DNA damage between the combination groups and stand-alone cisplatin treatment counterparts, suggesting a considerable synergism and better efficacy of cisplatin in combination with SMF, while on the contrary, such a synergistic effect of SMF was not noticeable in resistant groups. In both sensitive and resistant cells, the extent of damage increased gradually over time in the groups treated with the drug alone and in those receiving the combination.

The cell cycle can be arrested by the combination of chemotherapy drugs and magnetic field, and subsequently, cells will be prevented from undergoing mitosis; as a result, the cell population in the G2/M phase increases, whereas the cell population in the G0/G1 phase decreases^37,38^. ATM protein is a serine-threonine protein kinase, the expression levels of which increase due to damage caused by either the direct effect of drugs or free radicals generated by a magnetic field. It also activates regulatory proteins in the cell cycle, including E2F1, Cdc25, and p53 among others^39,40^. As represented in Figs. 6 and 7, all of the stand-alone cisplatin treatment and the combination groups from both cell lines were primarily arrested at the G2 phase; however, there were no significant distinctions between any of the included groups at this phase. Besides, the proportions of cells arrested at the G1 phase were significantly reduced in both stand-alone cisplatin and the combination groups although no tangible contrast was spotted between the reductions.

Some reports have demonstrated that the magnetic field may lead to a significant reduction in the levels of calcium ions in Bone marrow-derived stem/stromal cells, which can possibly decrease cell proliferation and increase the rate of apoptosis^41^. There have been several studies showing that the magnetic field may affect the homeostasis of iron atoms in certain cells and increase intracellular iron in the cytoplasm and nucleus, which gives rise to higher levels of hydroxyl radicals and as a result of apoptosis^39^. Apoptosis was found to be the predominant type of cell death following stand-alone cisplatin treatment, and the rate of cell death had a direct relationship with the duration of treatment. Cis + SMF at 96h in both cell lines and Cis + SMF at 48h from sensitive cells had significantly higher rates of apoptosis compared to both their control groups and the stand-alone cisplatin testament counterparts, which similar to the results of the comet assay proposed that the synergism of SMF with cisplatin might play a key role in increasing of cisplatin efficacy. Additionally, in the case of necrosis, combination groups from resistant cells, unlike the sensitive ones, had drastically higher percentages of cells undergoing necrosis compared to both their control group and stand-alone cisplatin treatment counterparts.

The results of real-time PCR showed changes in the expression levels of some of the studied genes (Fig. 10). The expression levels of the P53 gene, a well-known marker involved in apoptosis^42^, had increased in cis + SMF for 96h groups from both cell lines compared to both their controls and stand-alone cisplatin treatment counterparts, which were statistically significant, and similar to the previous findings support the positive effects of SMF in combination with cisplatin. The expression levels of the P21 gene were also affected by different treatments. Interestingly, the highest expression levels were observed in both resistant and sensitive groups treated with cisplatin combined with SMF for 96 h. Although these increases in expression level were statistically significant in comparison to their control, no significance was found between these combinations and their stand-alone cisplatin treatment counterparts.

In the case of the Bcl2 gene, one of the important anti-apoptotic proteins^43^, there were no substantial changes in expression levels among the treated groups. The results may suggest that cell death has been affected by the combination mostly through a mechanism of action independent of Bcl2. Several cancer studies have reported that higher expression levels of CTR1 are correlated with higher intracellular concentrations of pt-based compounds such as cisplatin and resultantly better prognosis following chemotherapy^44,45^. The analysis of the expression levels of the CTR1 gene showed the great effect of the combination on the expression levels of this protein. As evidenced by Fig. 10, treatment of cells with SMF or cisplatin alone had no significant effect on CTR1 expression levels. On the contrary, after the 96h treatment of cisplatin + SMF, the expression of this protein in both groups increased significantly compared to their controls and stand-alone cisplatin counterparts. Considering these results, the increase in the uptake of cisplatin by cells that were measured in our previous study^8^ can be interpreted as a crucial element for overcoming the resistance of the A2780cp cell line.

This study has some potential limitations. The incubator was handmade and its condition (Temperature, CO2, intensity of field in different areas of the chamber) should be monitored every day. However, if mistakes are made in monitoring these factors, it may affect the obtained results. There were many variables in this study, and this can complicate statistical analysis and interpretation of the results.

## Conclusion

Herein, our study demonstrates that the combination of cisplatin + SMF increased DNA damage in both sensitive and resistant cell lines resulting in higher rates of cell death, which was observed in both forms of apoptosis and necrosis. Besides, this combination increased the gene expression levels of P53 and P21, key genes involved in apoptosis, but had a limited impact on the expression levels of BCL2. Additionally, gene expression levels of CTR1 were significantly upregulated in groups treated with cisplatin + SMF. In conclusion, the present study suggests that the combination of SMF with other modalities such as cisplatin may show better anti-cancer efficacy and reduce the required doses of cytotoxic agents, hence lowering the risk of adverse effects; however, to translate SMF as a practical modality into the clinics, further investigations need to illuminate its effects on numerous aspects of tumorigenesis such as various pro-tumorigenic signaling pathways, other types of cell death e.g., autophagy and ferroptosis, cancer stemness, and angiogenesis, among many others both in vitro and in vivo.

## Data Availability

The datasets used and analyzed during the current study available from the corresponding author on reasonable request.
